# Transverse Circular Holes in Cylindrical Tubes Loaded in Traction and in Flexion: A New Analytical Approximation of the Stress Concentration Factor

**DOI:** 10.3390/ma13061331

**Published:** 2020-03-14

**Authors:** Alessandro U. Rebora, Gianluca Vernassa

**Affiliations:** 1 Department of Mechanical Engineering, Polytechnic School, University of Genoa, 16145 Genoa, Italy; 2 Polytechnic School, University of Genoa, 16145 Genoa, Italy; s4256627@studenti.unige.it

**Keywords:** concentration, hole, strength, stress, tension, tube

## Abstract

This paper presents an analytic formula for the theoretical stress concentration factor kt for cylindrical tubes with transverse circular holes, loaded in traction or in flexion. The study is based on modern finite element (FEM) techniques, which allow for appreciating with great accuracy the phenomenon of stress concentration. A comparison between the FEM results of this paper and those that were obtained by the existing analytic formulas shows the need of an update, as some discrepancies may be noticed. Our results are the fruit of a wide campaign of numerical FEM simulations that have been conducted analyzing numerous geometric configurations of the tube. Moreover, these configurations are defined in a wider two-dimensional (2-D) domain than the one valid for previous analytic formulas published in literature. Finally, these FEM results have been approximated with great accuracy by means of a fourth degree double polynomial regression that led to the new analytic formula that is proposed in this paper.

## 1. Introduction

Stress concentration phenomena occur whenever a structure charged with loads, which should normally cause a uniform stress distribution, presents strong stress gradients in a few localized very small areas instead.

If in proximity of the sudden variations of a mechanical component’s geometry, a regular stress distribution becomes very perturbed, and high stress peaks may be generated there. The risk is that, without an accurate evaluation of this aspect, a mechanical component or, more in general, a structure, may be quickly made to work beyond its design values, incompatible with the resistance of the material, thus compromising its functionality and safety.

Numerous examples of possible causes of stress concentration might be cited. Holes or notches machined in shell and plates loaded in traction, compression, or flexed, may certainly cause strong stress peaks. In the case of tubes, shafts, or, in general, in axisymmetric revolution bodies, besides the tensile and bending loads, which may cause peaks of the normal stress σ, the torsional load also induces a source of concentration for the shear stress τ. In order to quantify the effect that is produced by generic stress concentrators, a theoretical stress concentration factor kt is commonly defined as:

(1) $$kt=\frac{\sigma_{\max}}{\sigma_{n}}$$

In this formula σ_max_ stands for the maximum peak stress while σ_n_ is the so called “nominal stress”: the latter is a previously defined “reference” tension. For a pure torsional load, where the significant tension is the torsional stress, the same definition of kt applies to the shear stress τ, instead of to the normal stress σ.

The kt factor only depends on the geometry of the stressed elastic body and the type of external load to which the body has to resist. For this reason, the kt factor is also referred to as the geometric factor kg. Instead, the stress concentration factor is independent of the magnitude of the load or the material that the body is made of, as long as plasticity is not reached. Sometimes, kt is also referred to as the elastic factor ke, just because the behavior of the material is always supposed to remain linear and elastic, regardless of the level that is reached by the maximum tension.

The accurate determination of the stress concentration factor always requires proper and careful investigation. This can be carried out following two different approaches: through experimental techniques of structural investigation (such as, for instance, photoelasticity or extensometry), or by means of numerical calculations, which always provide approximate results, although the level of approximation can be improved more and more, thus reaching very high precision.

A few approximate analytical solutions that are expressed in closed form have sometimes also been calculated. One of the best known among them is certainly the solution that was found by Kirsch [1], about an elastic body provided with a small geometrical discontinuity and loaded in a uniform way. At the end of the nineteenth century (1898), he solved the problem of a thin infinite square plate with a small circular hole in its center, supposing the plate loaded by a traction or compression load, being uniformly distributed on two opposite sides of the plate.

However, excluding very simple geometries, such as perforated plates, the quest for an analytical solution written in a closed form is always a very difficult task, being often impossible to do. The use of other theoretical solution methods can also be considered. Nowadays the most known and diffused among them is the technique of finite elements (FEM) that has been developed since the sixties of the last century.

The current availability of powerful general-purpose structural software, being readily accessible and simple to use to develop accurate stress analyses, combined with a continuous increase in the performance of computer hardware, easy to purchase on the non-specialist market, has revolutionized this field of research. Consequently, nowadays, expensive and time-consuming experimental techniques have been practically replaced by FEM computational techniques, which are much more flexible and efficient, as well as much less expensive.

Through the years, several authors have published wide collections of elastic stress concentration factors valid for standardized geometries, both in the form of analytical formulas and in the form of families of curves or graphical charts, and then reproduced in numerous mechanical design books (e.g., Peterson, [2,3], Shigley and Mischke [4], Norton [5]). Nevertheless, nowadays, the need to increase the accuracy of kt factor evaluation is still present.

A sharp determination of the stress concentration factor value is in fact a basic step for the correct design of machine parts or mechanical components made of brittle materials undergoing static constant loads and, especially, for ductile materials that are stressed by recursive cyclic loads that induce fatigue damaging. It has indeed been estimated that an error of about 10% for the stress concentration factor could lead to a factor of 5 or greater error in expected fatigue life.

Although the calculation of a kt factor todays appears to be a well-known and widely examined problem for plenty of geometries, the particular case that we have studied in this paper is not so diffusively treated, either in mechanical design books, or in articles published in literature. Anyway, one of the most known among them is surely the work of Jessop, Snell, and Allison [6] published in 1958, which is based on the experimental results that were obtained by means of the photoelasticity method. Jessop’s work might be considered as an improved evolution of the researches presented in previously published papers [7,8,9,10], all being developed on the base of experimental techniques.

The first publication where FEM analyses are used to validate experimental results is an ESDU work executed in 1989 [11]. It should be highlighted that, until that year, all of the commercial finite element codes could treat with great practical difficulties, so complex three-dimensional (3-D) geometries and certainly using a limited number of elements, because of the low computational power of the available hardware of that time. Additionally, a recent book [12] considers the ESDU work of 1989 as an up to date FEM analysis. For this reason, we believe that a new investigation that was developed on the base of an accurate and validated parametric model, while using the current version of a widespread FEM code might lead to somewhat different numerical results and surely more precise.

The intent of this paper is to formulate a new polynomial expression of the kt stress concentration factor, being extended to a polynomial’s degree that is higher than that used in [12,13] and able to cover a two-dimensional (2-D) domain of geometries, which is a bit wider than the previous ones. This new polynomial form is the fruit of a regression technique of the obtained FEM results. Additionally, this second task is very delicate and must be conducted with great care to avoid perceptible differences between the original FEM data points and the values that were obtained by using the polynomial expression.

In the next paragraph, we present the method followed to construct and analyze a parametric FEM model able to describe a family of different geometrical configurations loaded in traction or in flexion, all being recursively generated from the same basic topology. The following paragraph deals with the FEM results that were obtained from recursive analyses and the subsequent careful numerical elaboration (double polynomial regression technique) that allow for the formulation of the new expression of kt here presented. Finally, our results are submitted to a critical discussion to assess the correctness and the intrinsic value of our proposal.

## 2. Materials and Methods

Several equivalent tools may be used to conduct accurate linear FEM analyses (ABAQUS, ALGOR, Lusas, MSC Nastran, Straus 7, etc.). The code at our disposal is probably the most diffused in the world: ANSYS APDL [14]. Additionally, the double polynomial regression might be handled by means of different tools. In particular, we have chosen the program MATLAB R2019b [15], since it permits a graphic representation of undulating 3-D surfaces, which is judged to be optimal.

**Figure 1:**
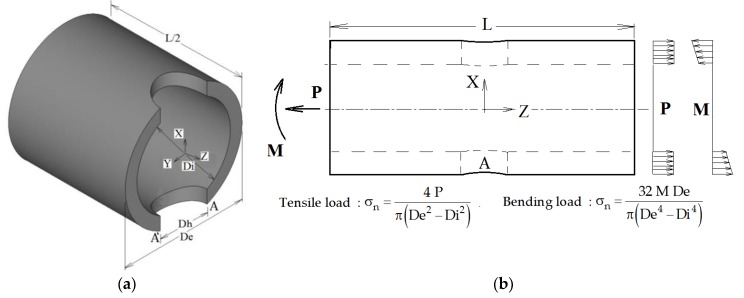
(**a**) Characteristic dimensions of the geometric configuration; and, (**b**) Distributions of applied loads.

The wide campaign of surveys that we have performed by numerical FEM simulation considers the geometry shown in the simple qualitative sketch of the structure analyzed, which is depicted in Figure 1a,b. Figure 1a is an axonometric view of the geometrical 3-D model that highlights three characteristic dimensions: (i) the external tube diameter De; (ii) the internal tube diameter Di; and, (iii) the hole diameter Dh. The tube length L is not considered as a characteristic dimension: in our analyses, the tube was modelled long enough to fulfil Saint–Venant’s Principle, while, in practical use of the analytical formulation, it is up to the designer to evaluate other local effects that may occur. Figure 1b is a front view of the tube showing the applied loads (a uniform traction P or a bending moment M). 

In particular, the traction (or compression) load P is applied as a constant surface load acting on the tube cross sections placed at z = ±L/2. The bending moment M acts on these cross sections of the tube as a surface load distribution, having zero resultant force and a constant X slope.

The three characteristic dimensional quotes De, Di, and Dh may completely define the geometric shape of every FEM calculation model analyzed. Accordingly, if we choose an arbitrary constant value of the tube external diameter De, only two independent non-dimensional factors (the ratios Di/De and Dh/De) are required for defining any particular geometric model from which the full 3-D FEM model (made of solid brick and pyramid elements) is created, by means of the automatic mesh generation technique that is used in ANSYS.

The stress concentration factor kt is evaluated using the maximum FEM σ_z_ stress that develops at points A–A’, which were placed at the intersection between the hole contour and the X-Y symmetry plane. The nominal stress σ_n_ is evaluated according to the beam classic theory (see the formulas shown in Figure 1b).

So doing, the stress concentration factor kt might be seen as an implicit and unknown function of two non-dimensional factors: the “thinness factor Ft” expressed by the ratio Di/De and the “hole factor Fh” expressed by the ratio Dh/De.

The parameters Ft and Fh cannot assume any positive value, but they should respect the two range limits: 0.59 = Ft_min_ ≤ Ft ≤ Ft_max_ = 0.98 and 0.05 = Fh_min_ ≤ Fh ≤ Fh_max_ = 0.54.

We have chosen 0.54 = Fh_max_ < Ft_min_ = 0.59, as these values come from the geometrical condition Dh_max_ < Di_min_, which must be widely satisfied to prevent any geometric shape incompatibility, even when the largest hole diameter Dh is combined with the thickest tube (i.e., together with the smallest internal diameter Di) approaching too closely the limit condition of tangency between the longitudinal cylindrical cavity of the tube and the transverse hole. On the other hand, the limit Ft ≤ 0.98 has been fixed to exclude too thin tubes from our analyses.

Both of the ranges have been divided into n = 25 intervals, obtaining n + 1 = 26 “notches”, i.e., end points of each interval. The amplitude A_i_ of each i-th interval is not constant, as the sequence of intervals has been chosen, such that A_i+1_ = r A_i_ = $\sqrt[{n-1}]{q}$A_i_ (with q = A_n_/A_1_), therefore composing a geometrical progression. If q > 1 the sequence of intervals is denser near the first end of the range and less dense near the second end of the range (the contrary holds if q < 1). We have fixed q = 3 = qt, while for the polarization in the Fh variation range we have fixed q = 1/3 = qf, in order to polarize the density of intervals of the Ft variation range.

A matrix of 26 × 26 = 676 calculation points has been processed, uninterruptedly analyzing 676 different geometries generated by an ANSYS APDL algorithm that we have implemented. In this algorithm, two nested do loops recursively modify the Ft and Fh values and, consequently, the characteristic dimensions of the i-th current geometric model.

The first step of every FEM analysis consists in the choice of element type, order of integration, and mesh density. Our model is mainly composed of second order 20-node solid hexahedral elements (SOLID 186 of ANSYS Element Library). A pyramid-shaped 13-node solid element might also be obtained by collapsing the eight nodes of a quadrilateral face into one single node, which becomes the vertex of the pyramid. We have used this particular element shape to correctly mesh a transition volume interposed between two adjacent volumes both filled with semi-mapped mesh of hexahedral elements having different size. We have also adopted a 2 × 2 × 2 full integration scheme of Gauss points.

The element size has been chosen to be small enough around the peak point A of Figure 1a, although it becomes larger going away from that point. A mesh sensitivity study for each of the 676 different geometry is obviously useless, as well as practically impossible to achieve. Thus, we have only studied four limiting cases corresponding to the four corner points of the 2-D domain resulting from intersection between the Ft range and Fh range: (1) Ft = Ft_min_, Fh = Fh_min_; (2) Ft = Ft_min_, Fh = Fh_max_; (3) Ft = Ft_max_, Fh = Fh_min_; (4) Ft = Ft_max_, Fh = Fh_max_.

**Figure 2:**
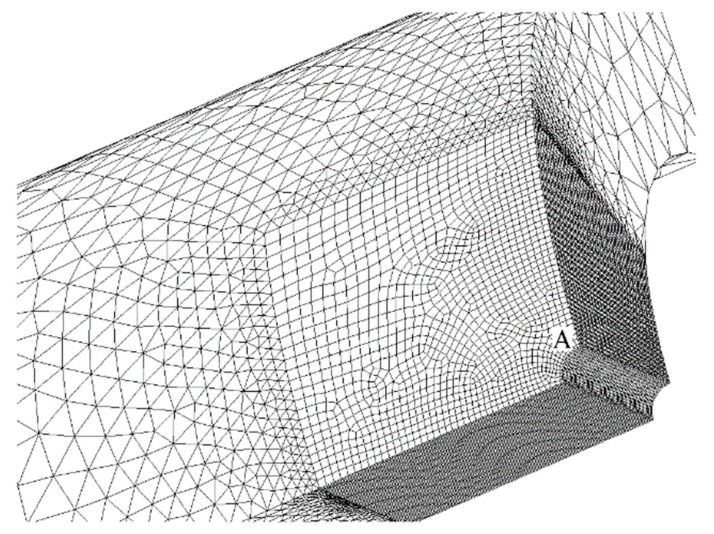
The finite elements (FEM) mesh of case 1.

**Figure 3:**
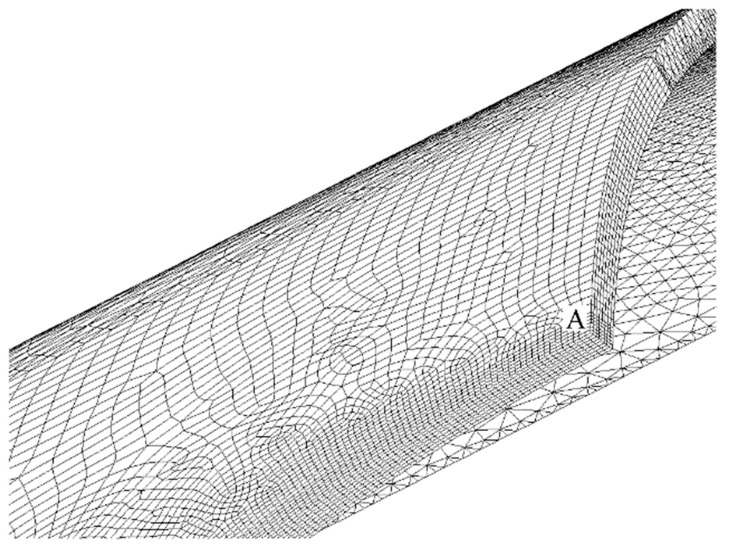
The FEM mesh of case 4.

Figure 2 and Figure 3 present, respectively, the mesh generated in cases 1 and 4 (the most significant ones) zoomed near the peak point A. The model only reproduces one-eighth of the tube, thanks to: (i) triple symmetry of boundary conditions for load case P; and, (ii) symmetry/asymmetry of boundary conditions for load case M (symmetry on planes X-Y and Z-X, asymmetry on plane Y-Z).

**Figure 4:**
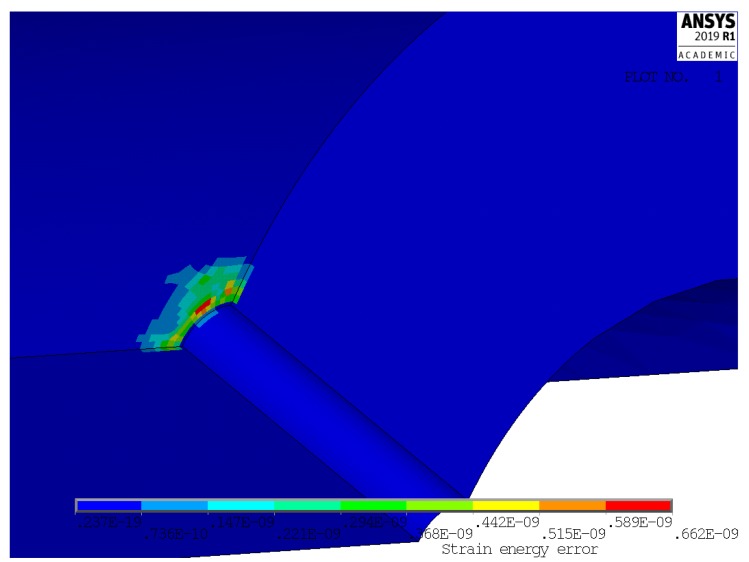
Structural error energy in case 1.

**Figure 5:**
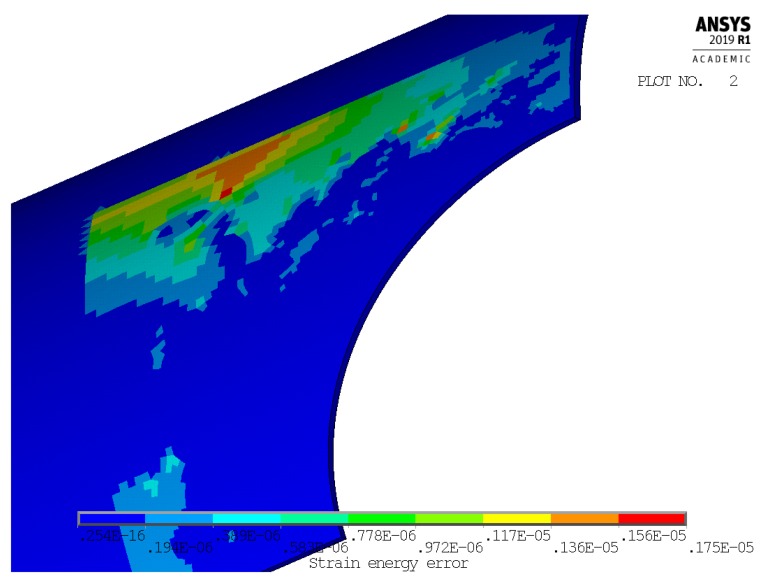
Structural error energy in case 4.

Figure 4 and Figure 5 show, instead, a plot of the structural error energy for cases 1 and 4, being observed from a view point a bit different than before.

Several books treat the criterion of the strain energy error [16,17] and widely applied. The values of the structural percentage error in energy norm (SEPC) corresponding to the error energy plotted in Figure 4 and Figure 5 are both lower than 2.5%. This value is under the conventional limit that addresses the quality of mesh in the local area of high stress. This is sign of an optimal mesh size in both of these significant cases. Furthermore, better results are found in cases 2 and 3.

## 3. Results

**Figure 6:**
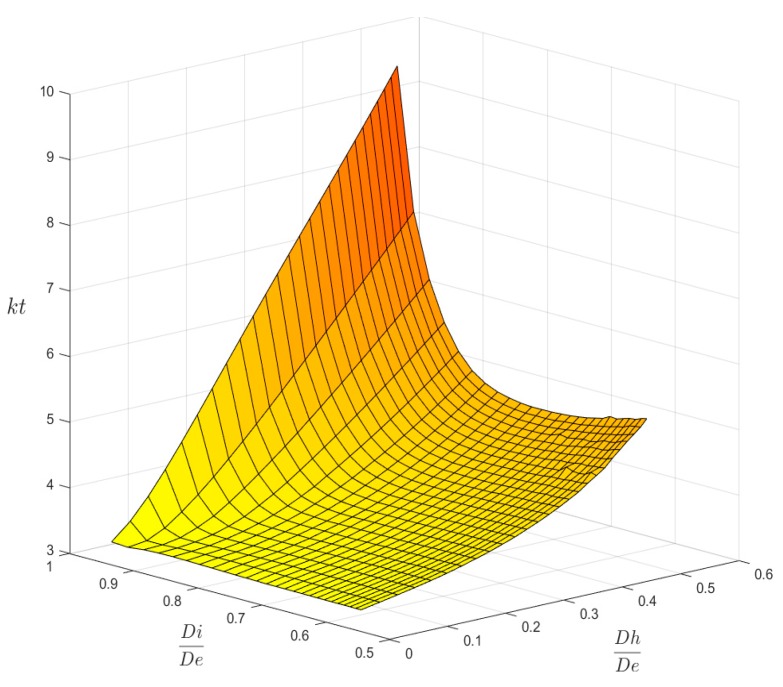
The kt surface for P load.

**Figure 7:**
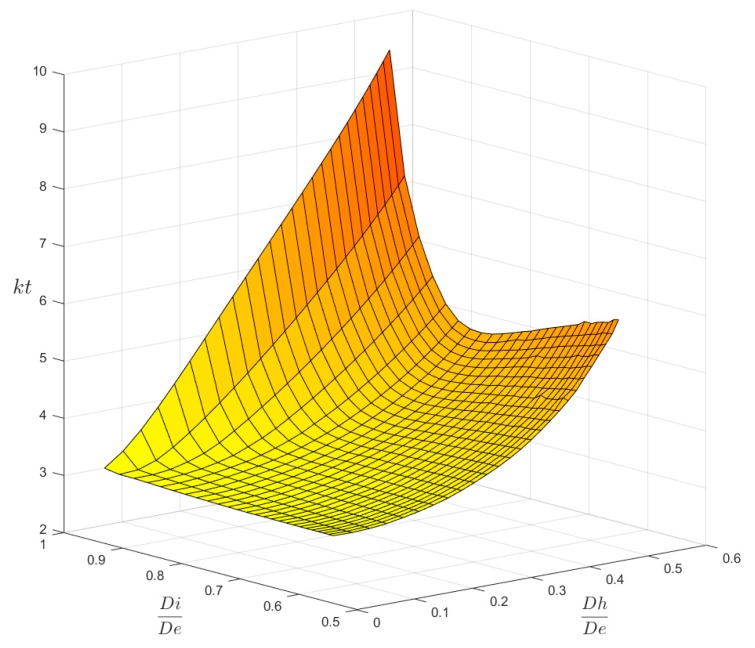
The kt surface for M load.

Figure 6 and Figure 7 show the main result that was obtained at the end of the FEM simulation campaign. In Figure 6 the 3-D surface of kt values is plotted for axial load P, while Figure 7 shows the surface that was obtained for bending load M. Both surfaces are based on a total of 26 × 26 = 676 calculation points.

**Figure 8:**
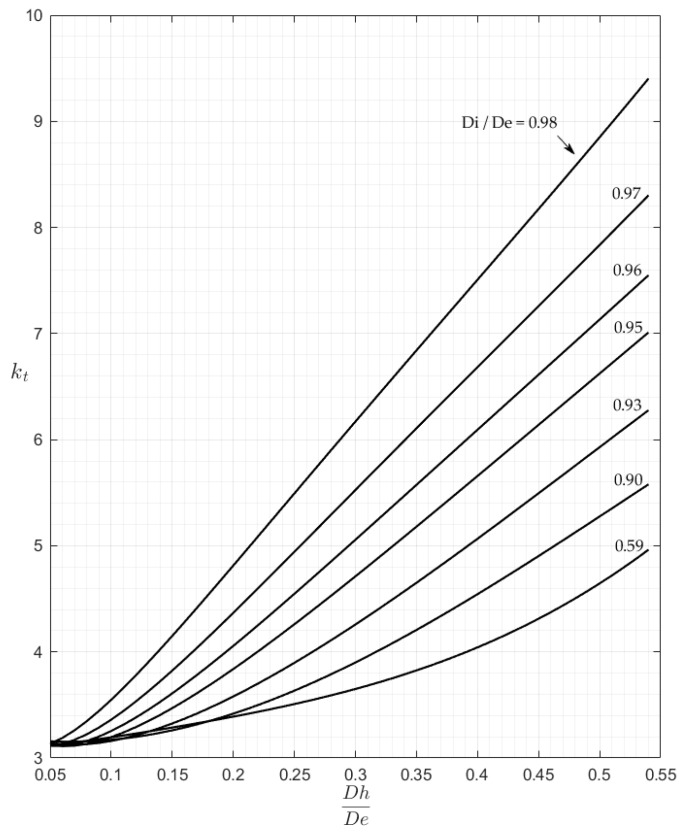
The kt chart for P load.

**Figure 9:**
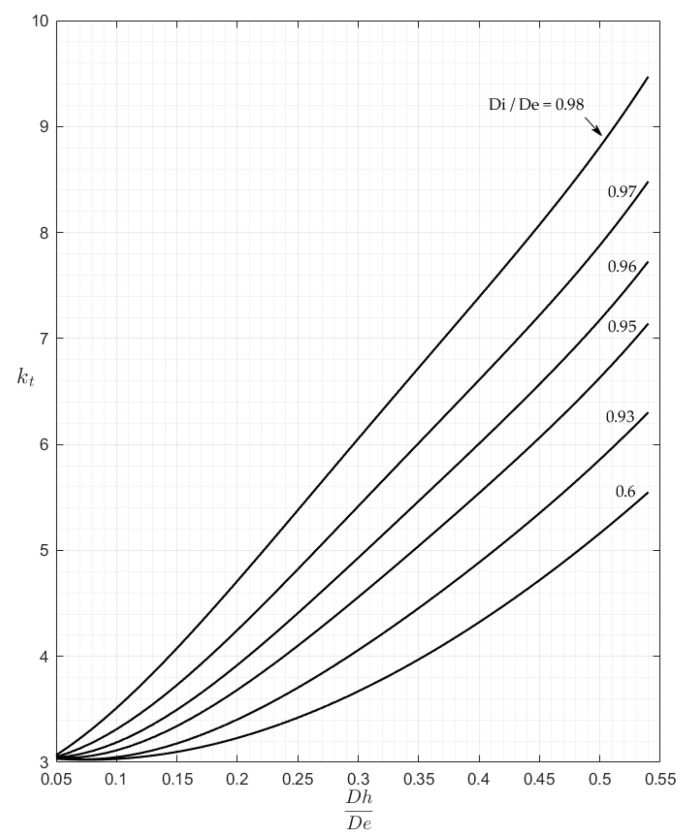
The kt chart for M load.

These 3-D plots are less practical for the rapid evaluation of the kt factor than traditional charts which use an entry point chosen in the abscissa axis. However, any 2-D chart might be easily obtained just by intersecting the surfaces of Figure 6 and Figure 7 with a family of vertical planes that may be orthogonal either to the Ft axis, or to the Fh axis. Figure 8 and Figure 9 show, as an example, the charts that were obtained for P and M load, while assuming the Fh parameter as the x-axis variable and using seven intersection planes in Figure 8 and six intersection planes in Figure 9.

After completing the campaign of FEM analyses, the subsequent double polynomial regression technique has allowed for the formulation of this new analytic approximation formula for calculating the stress concentration factor kt:

(2) $$\begin{array}{c} kt=C_{0}+C_{1}{\left(\frac{Di}{De}\right)}^{a}+C_{2}{\left(\frac{Di}{De}\right)}^{2a}+C_{3}{\left(\frac{Di}{De}\right)}^{3a}+C_{4}{\left(\frac{Di}{De}\right)}^{4a}\\ (a=\;9.2\;\mathrm{for}\;P\;\mathrm{load};\;a=7.6\;\mathrm{for}\;M\;\mathrm{load}) \end{array}$$

Every C_i_ coefficient is, in turn, the result of another polynomial expression:

(3) $$\begin{array}{c} C_{j}=\alpha_{j0}+\alpha_{j1}{\left(\frac{Dh}{De}\right)}^{\frac{1}{b}}+\alpha_{j2}{\left(\frac{Dh}{De}\right)}^{\frac{2}{b}}+\alpha_{j3}{\left(\frac{Dh}{De}\right)}^{\frac{3}{b}}+\alpha_{j4}{\left(\frac{Dh}{De}\right)}^{\frac{4}{b}}\\ \;(b=1.55\;\mathrm{for}\;P\;\mathrm{load};\;b=0.952\;\mathrm{for}\;M\;\mathrm{load}) \end{array}$$

The numerical results of the regression process are shown in Table 1, where a square (5 × 5) matrix is filled with 25 α_ij_ coefficients that were calculated for the case of an axial load P, while Table 2 presents the matrix for a bending load M.

If we define the thinness column-vector $\left\{Fta\right\}=\left\{Ft^{0},Ft^{a},Ft^{2a},Ft^{3a},Ft^{4a}\right\}$ and the hole column-vector $\left\{Fhb\right\}=\left\{Fh^{0/b},\;Fh^{1/b},\;Fh^{2/b},\;Fh^{3/b},\;Fh^{4/b}\right\}$, the synthetic calculation providing the polynomial approximation of the stress concentration factor might be simply expressed by the matrix product:

(4) $$kt={\left\{Fta\right\}}^{T}\left[\alpha \right]\;\left\{Fhb\right\}$$

This compact form is very useful to compile a simple computer code able to provide a kt value calculated as the result of three input values: the tube diameters De, Di, and the hole diameter Dh. In the Appendix A, the listing is shown of an example code (script), written in MATLAB language, which can be used to immediately calculate the theoretical stress concentration factor of a cylindrical tube with transverse circular hole loaded in traction or in flexion.

## 4. Discussion

**Figure 10:**
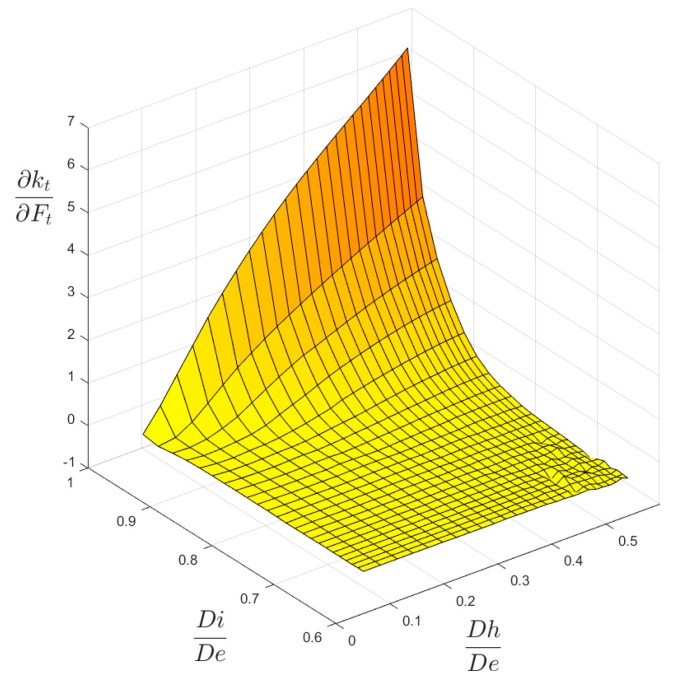
The ∂kt/∂Ft surface for P load.

**Figure 11:**
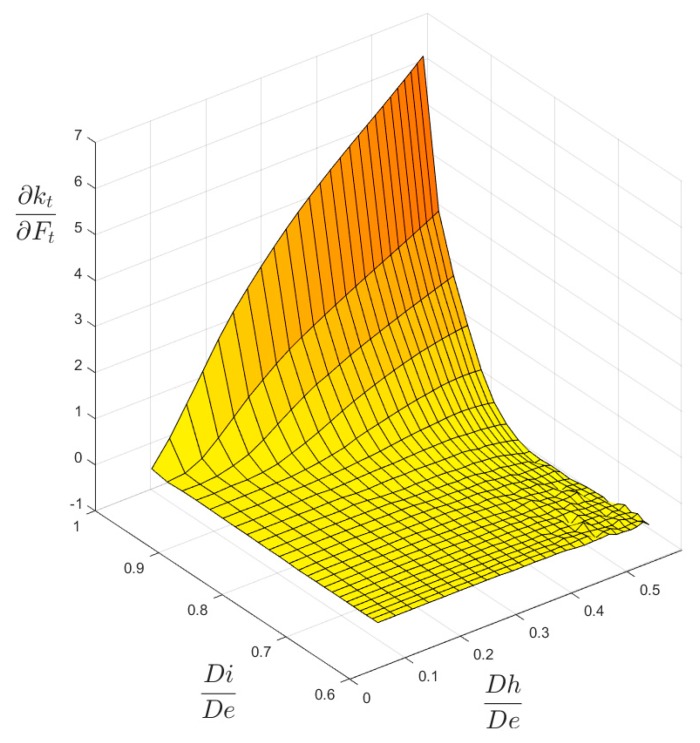
The ∂kt/∂Ft surface for M load.

Another important result that, besides Figure 4 and Figure 5, validates the chosen local mesh density, not only in the four corner points of the 2-D domain of geometric configurations, but also all over it, is shown in Figure 10 and Figure 11. These two figures present a 3-D plot of the numerical partial derivative ∂kt/∂Ft for load types P and M, respectively. Both surfaces are very “smooth” and no significant relative maximum (or minimum) is evidenced. Similar results are found for the partial derivative ∂kt/∂Fh. This is sign of a satisfactory continuity of the kt surface and it confirms the good quality of the element mesh all over the 2-D domain.

Moreover, the mapped meshes that are plotted in Figure 2 and Figure 3 show an optimal aspect ratio near point A (very near to 1), together with an angle between any of two adjacent edges of the element that is equal (or almost equal) to a right angle. Beside the high mesh density, an optimal value of these two parameters also contributes to the best quality of the results.

**Figure 12:**
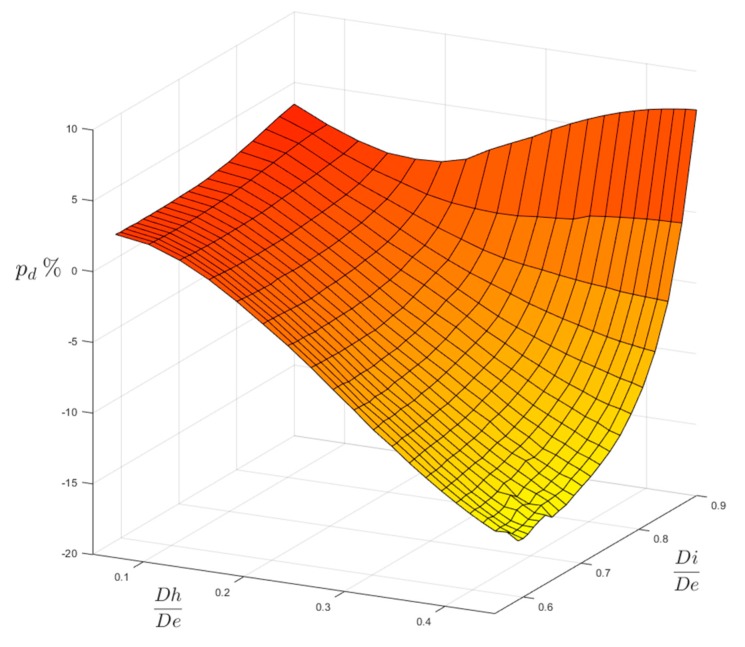
Surface of percentage differences for P load.

**Figure 13:**
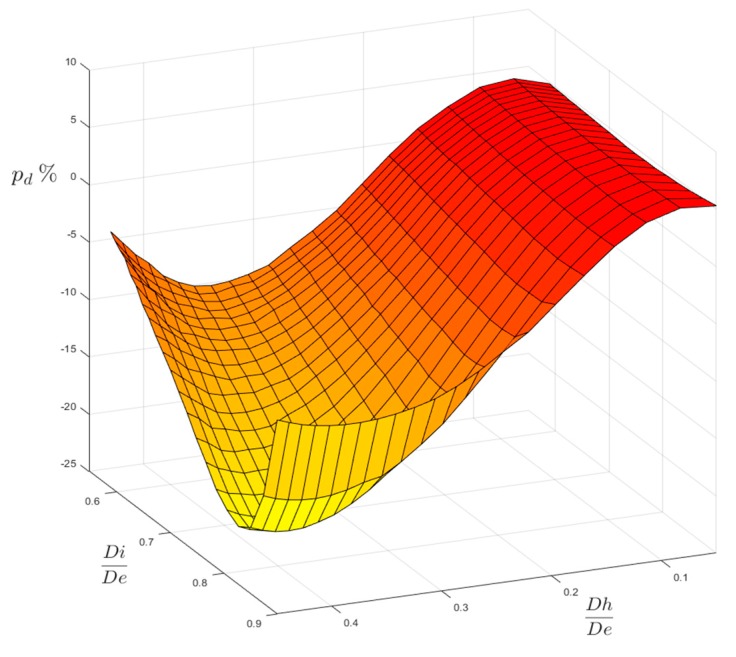
Surface of percentage differences for M load.

However, another comparison may confront our FEM results with the ones evaluated according to the existing formulas that were checked by ESDU and published in [12], as shown in in Figure 12 and Figure 13.

The plotted surface of both figures represents the percentage differences p_d_ evaluated with respect to our FEM-based results:

(5) $$p_{d}=100\times \frac{kt_{FEM}-kt_{ESDU}}{kt_{FEM}}$$

where p_d_ > 0, our FEM results are more conservative than the ESDU ones; where p_d_ < 0, the ESDU formulas provide more conservative results instead. The variation ranges of Figure 12 and Figure 13 are a bit different than those that were previously used in Figure 6 and Figure 7 (0.59 ≤ Ft ≤ 0.98; 0.05 ≤ Fh ≤ 0.54). The extension of the domains of Figure 12 and Figure 13 has been indeed reduced by lowering the Ft upper limit (Ft ≤ 0.90, both for P load and for M load) and the Fh upper limit (Fh ≤ 0.45 for P load; Fh ≤ 0.40 for M load), since the formulas that are reported in [12] are not valid either for very thin tubes (Ft > 0.90) or very large holes (Fh > 0.45 for P load and Fh > 0.40 for M load).

The comparison gives quite different results, depending on the type of load. For load P, the agreement between FEM and ESDU results is good in the region of small holes, whatever the thickness of the tube. In the region of large holes made on thicker tubes our results are less conservative, as they provide lower values of the stress concentration factor (lower by −15.79% as a minimum), but, when large holes are made on thinner tubes, our results are more conservative (greater by 7.24% as a maximum). In the case of load M, the agreement is, in general, slightly less good then for P load. Indeed, a minimum negative difference of −20.56% occurs for large holes made on moderately thin tubes. On the other hand, a maximum positive difference of 5.75% occurs for small holes that were made on thinner tubes.

In Figure 6 and Figure 7, we can observe some small undulations of the surfaces traced in the area corresponding to the large holes made on the thick tubes, a sign of a sort of disturbance in the distribution of the FEM results when compared to the regular trend clearly evident in the other areas.

We have noticed that adopting different spacing criteria for the parameters Ft and Fh (i.e., different values of the parameters qt and qf) affect the subsequent regression operations, giving worse results in terms of adherence of the analytical model to the numerical one. However, all of these aspects will be treated in greater detail, only after introducing the following Equations (6)–(9).

The examination of Figure 8 shows an asymptotic value of about 3 for all of the curves corresponding to various Ft values. This result indicates that, for very small holes, the theoretical solution found in [1] for plane plates also applies to cylindrical tubes, whichever the thickness of the tube.

Some other considerations are now made regarding the limiting extension of the calculation models and, in particular, the axial length L of the tube model that has been fixed large “enough” to fulfil Saint-Venant’s Principle in every cyclic FEM model analyzed during the i-th loop. The value assigned to the tube length L has been systematically verified during the calculation loops, since the implemented APDL algorithm controls the displacement distribution obtained on the tube ends (when a uniform tensile distributed load P is applied) was itself always uniform with a percentage difference from its mean value of less than 0.5%. For a flexion load M, the presence of a uniform distribution (with the same previous admissible percentage difference) has been checked for the bending rotation angle around the Z-axis shown in Figure 1a, being calculated by means of the z-displacements of the nodes lying on the tube’s end sections.

Further information regarding the execution of numerical calculation is now given to roughly quantify the problem size, the computational times, and the characteristics of the hardware used. In particular, the number of second order SOLID186 elements composing a generic current model may vary during cycles within the approximate range 17,000–152,000, while the number of nodes varies in the approximate range 56,000–460,000. The total elapsed computer time, spent to perform a set of 767 calculations, is about 3.6 h, running on our commercial 10-core 64 GB RAM desktop Dell Xeon workstation with 3.3 GHz base frequency.

Many are the analytical expressions that were proposed in the past for the calculation of stress concentration factors in various configurations. Choosing one among them that would be adequate to approximate the FEM surfaces was a crucial point of this work. Hence, every choice made was based on evaluations of two fundamental parameters that assess the regressions.

The first of them is the maximum (among the 676 values of the grid) percentage difference δ between the FEM results and the proposed analytical model:

(6) $$\delta =100\times \frac{kt_{FEM}-kt_{Anal}}{kt_{FEM}}$$

The second parameter is the “determination coefficient”, which is indicated in the statistical literature as the parameter r^2^. This is widely used in probability calculations and, although there is no univocal mathematical expression of its meaning, we can generally say that it quantifies the adequacy of the analytical model to describe the variability of the data. In the formulation that we have adopted, the coefficient of determination is simply the square of the correlation coefficient:

(7) $$r^{2}=\frac{S_{t}-S_{r}}{S_{t}}$$

The symbol S_t_ stands for the variance of the FEM data, while symbol S_r_ is the mean squared error (MSE) that results from the adopted polynomial model:

(8) $$S_{t}=\frac{1}{N}{\sum_{i=1}^{N}\left(y_{i}-\bar{y}\right)}^{2}$$

(9) $$S_{r}=\frac{1}{N}{\sum_{i=1}^{N}\left({\hat{y}}_{i}-\bar{y}\right)}^{2}$$

According to the above formulation, the parameter r^2^ can only assume values contained nside a range going from zero to the unit value, limits, which represent, respectively, an inadequate model returning the only mean value of the data (for r^2^ = 0) or an optimal model, capable of representing all of the values assumed by the data in a certain domain (for r^2^ = 1).

However, we should remember that such a result, r^2^ = 1, can always be obtained with a polynomial expression whose degree is equal to the number of data points for which the regression is conducted. This procedure is not advisable, as not only does the number of monomials grow with the number of values, but it also does not take the intrinsic uncertainty of the results into account.

As mentioned above, for this geometry a polynomial was chosen, based on the previous formulas that were published in [12,13]. In particular, the formulas of [12] for load P include a third degree polynomial in terms of Ft and a second degree one for Fh, while, for load M, it includes a second degree polynomial in terms of Ft and a third degree one for Fh. On the contrary, the choice followed by the authors of [13] always include (both for P load and for M load) a third degree polynomial in terms of Fh and a second degree one for Ft.

Our starting point were the formulas of [12], which respect to the formulas of [13] admit a larger Fh domain for the M load. However, when conducing regressions with the least square method, the results in terms of δ and r^2^ showed a bad correspondence between the FEM results and the new analytical ones. Hence, some changes were necessary in the original formulation.

First, the polynomial expressions for Fh and Ft were both brought to the fourth degree, already showing improvements for both δ and r^2^ but still not sufficient. Accordingly, the investigation in terms of the exponents *a* and *b* was carried out, and a multi-objective optimization problem occurred. Values of *a* and *b* that would minimize δ were not coincident with the ones maximizing r^2^.

**Figure 14:**
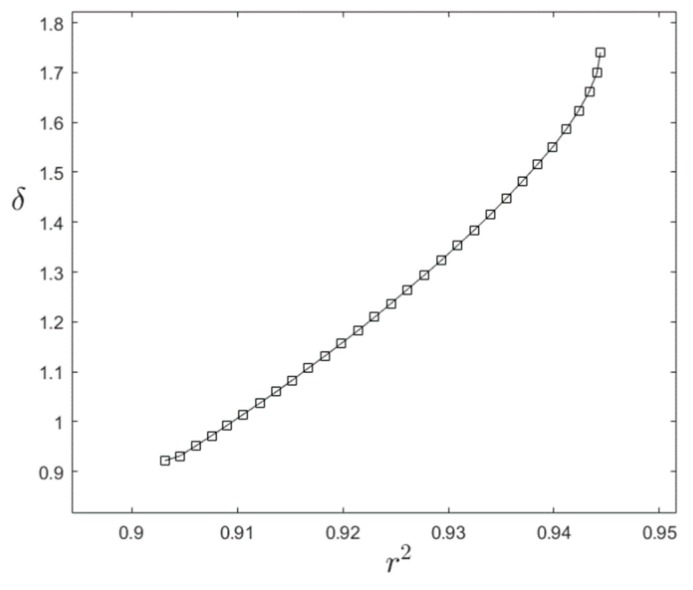
Pareto front for optimizing δ or r^2^ with load P.

Figure 14 shows the Pareto front of our problem for the case of axial load P, obtained by varying the exponents *a* and *b* between the values that would optimize either δ or r^2^.

As the quantities δ or r^2^ are related to each other (although in a complex way), we chose, for both loads P and M, the coefficients *a* and *b* that would maximize the determination coefficient, as this last one measures the overall trend of the results better. With this choice we were able to accomplish values of r^2^ = 0.94 for load P, and r^2^ = 0.99 for load M, signs of a near perfect adherence of the analytical model to the numerical results, as r^2^ almost reaches unity.

Afterwards, we investigated the nature of the consequent δ’s. This showed that the maximum δ’s. (δ = −1.73% for load P and δ = −1.01% for load M) always occur when Fh = 0.54 and Ft = 0.59, so in very localized corner of our definition domain. At the same time, we obtain the average error of 0.25% for load P, and 0.26% for load M while considering the mean value of all the percent differences (in fact, their absolute value), hence confirming the strong adherence between the two, analytical and numerical models.

## 5. Future Developments of Analysis and Conclusions

The main result that was obtained at the end of this research consists of a new polynomial expression providing the stress concentration factor kt valid over definition ranges for Di/De and Df/De that are a little wider than those that were admitted in the formulas available in literature. In particular, our analyses revealed that stresses were perceptibly overestimated by ESDU formulas, as much as 20% for some geometries and types of load. If using the new formulation here proposed, the consequent oversizing of a few parts in preliminary design stage of such notched structures might then be avoided.

However, our work is not concluded here, and further developments may be envisaged. The points A–A’ placed in Figure 1b on the line segments resulting from the intersection between the hole’s contour surface and the X-Y symmetry plane are those points where a stress peak occurs, as already stated. Obviously, a third coordinate giving the position of the peak’s point along the tube thickness should be used.

Although the nominal stress used for load M reaches its maximum value at the tube outer surface, while the nominal stress used for load P is constant across the tube thickness, the maximum FEM stresses are still found in a different position [18], which depends on the tube thickness and the hole amplitude, while the type of load (P or M) exerts a minor influence.

**Figure 15:**
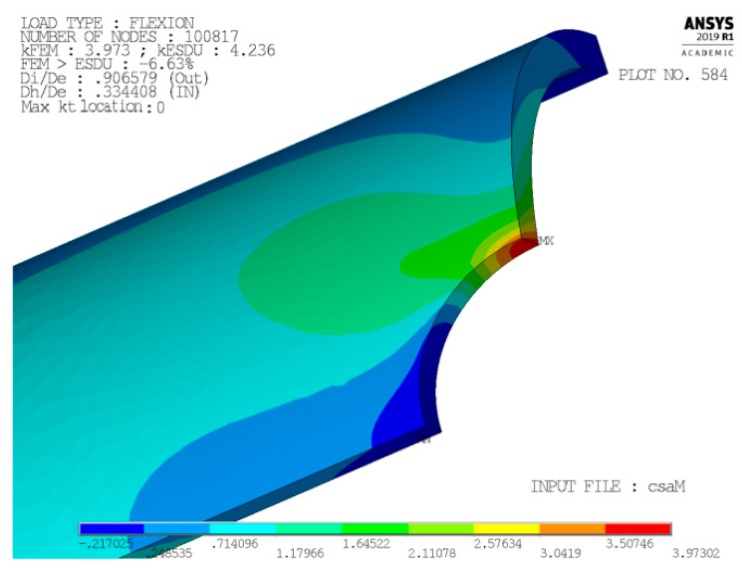
Large holes made on thin tubes.

**Figure 16:**
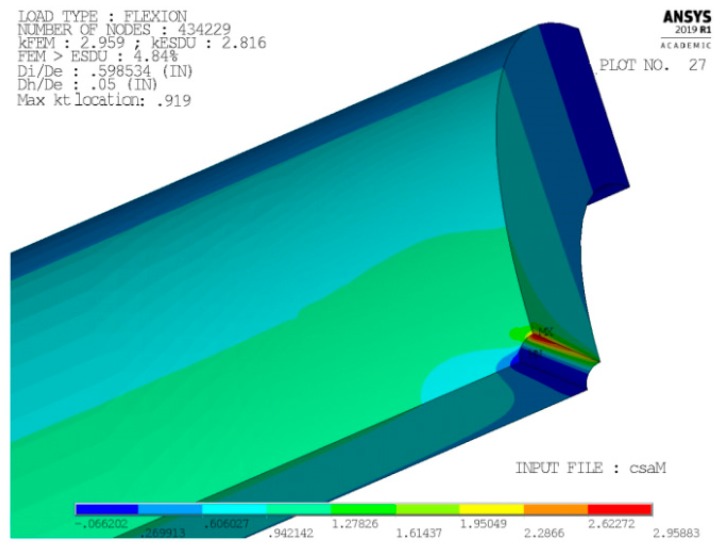
Small holes made on thick tubes.

In particular, we found that for load M the peak’s position lies on the tube’s inner surface when large holes are combined with small values of tube thickness. On the contrary, when small holes are made on the thickest tubes, the peak’s position tends to the tube’s outer surface, however without ever reaching it (see, for instance, Figure 15 and Figure 16).

A non-dimensional parameter ρ that is able to localize the position of the peak’s point might be defined as the ratio:

(10) $$\rho =\frac{Dp-Di}{De-Di}$$

where Dp is twice the radial coordinate of the peak’s point. In Figure 15, the ρ value is 0 (i.e., Dp = Di), while, in Figure 16, the ρ value is 0.919, indicating a peak’s point that lies near the tube’s outer surface.

If we consider the geometrical models of Figure 15 and Figure 16 subjected to a load P instead of M, similar ρ values are found, although not being exactly the same as those that were obtained for the load M. However, just for technical purposes, the peak’s point position might be considered independent of the load type. Assuming then this approximation, a simple formula (weighed mean) might be proposed to express the stress concentration factor kt in the case of a combined load P + M:

(11) $${\mathrm{kt}}_{\mathrm{PM}}=\frac{\sigma_{nP}{\;\mathrm{kt}}_{P}+\sigma_{nM}{\;\mathrm{kt}}_{M}}{\sigma_{nP}+\sigma_{nM}}$$

where σ_nP_ and σ_nM_ are the nominal stresses that are reported in Figure 1b, while ${\mathrm{kt}}_{P}$ and ${\mathrm{kt}}_{M}$ are calculated by means of Equation (4), using, for instance, the MATLAB code listed in the Appendix A.

This combination formula (as already proposed in [12], although in the form of Equation 4.147) needs, however, to be verified by comparison with the FEM results that were obtained for a generic model loaded with a combined external load given by the sum P + M. That might be accomplished with a “casual” spot approach, or, more rigorously, by means of a systematic approach that is based on a campaign of numerical simulations much wider than the one here presented.

For this second aim, we are indeed planning to conduct other similar recursive FEM calculations, by increasing the number of nested do loops included in the ANSYS APDL algorithm we have implemented. A new external loop controlling the recursive load M value and a further and more external loop controlling the recursive load P value will be added to the current double nested do loops, thereby greatly increasing the required computer elapsed times. If 25 intervals are fixed again in the new ranges chosen for P and M load values, the number of calculations to be executed will jump from the present 676 different FEM analyses to the huge number of 676 × 676 = 456,976. However, we expect to boost our computational resources today available and such a future challenge now appears to be feasible with success.

## Figures and Tables

**Table 1:** The α_ij_ coefficient matrix for P loading.

3.21773 × 10^0^	−2.33368 × 10^0^	1.54836 × 10^1^	−3.13707 × 10^1^	2.88392 × 10^1^
−2.29023 × 10^0^	3.38964 × 10^1^	−1.63629 × 10^2^	3.16564 × 10^2^	−2.35519 × 10^2^
2.56195 × 10^0^	−6.06259 × 10^0^	−6.40708 × 10^1^	1.97991 × 10^2^	1.18634 × 10^1^
1.49226 × 10^1^	−2.62897 × 10^2^	1.25373 × 10^3^	−2.16124 × 10^3^	9.97523 × 10^2^
−1.64984 × 10^1^	2.44015 × 10^2^	−1.01754 × 10^3^	1.64814 × 10^3^	−7.75593 × 10^2^

**Table 2:** The α_ij_ coefficient matrix for M loading.

3.04406 × 10^0^	−2.69793 × 10^0^	2.25618 × 10^1^	−4.30904 × 10^1^	5.71923 × 10^1^
−1.87267 × 10^−1^	1.03201 × 10^0^	4.57710 × 10^1^	−2.57433 × 10^2^	1.83344 × 10^2^
−1.13390 × 10^0^	9.84850 × 10^1^	−1.00954 × 10^3^	3.32593 × 10^3^	−2.89630 × 10^3^
7.22977 × 10^0^	−3.46562 × 10^2^	2.87425 × 10^3^	−8.16148 × 10^3^	6.93255 × 10^3^
−7.05391 × 10^0^	2.81564 × 10^2^	−1.99672 × 10^3^	5.25313 × 10^3^	−4.34765 × 10^3^

## Appendix A

% ===================================================================

% ************** MATLAB CODE FOR THE EVALUATION OF Kt **************

% ===================================================================

close all

clear all

clc

err = 1;

while err == 1

De = input(‘Insert the external diameter = ‘);

Di = input(‘Insert the internal diameter = ‘);

Dh = input(‘Insert the hole diameter = ‘);

load = input(‘Choose the load, press [1] for P, [2] for M: ‘);

Fh = Dh/De;

Ft = Di/De;

KP = [3.21773, -2.33368, 1.54836e+01, -3.13707e+01, 2.88392e+01;...

  −2.29023, 3.38964e+01, -1.63629e+02, 3.16564e+02, −2.35519e+02;...

  2.56195, −6.06259, −6.40708e+01, 1.97991e+02, 1.18634e+01;...

  1.49226e+01, −2.62897e+02, 1.25373e+03, −2.16124e+03, 9.97523e+02,;...

  −1.64984e+01, 2.44015e+02, −1.01754e+03, 1.64814e+03, −7.75593e+02];

KM = [3.04406, −2.69793, 2.25618e+01, −4.30904e+01, 5.71923e+01;...

  −1.87267e-01, 1.03201, 4.5771e+01,−2.57433e+02, 1.83344e+02;...

  −1.1339, 9.8485e+01, −1.00954e+03, 3.32593e+03, −2.8963e+03;...

  7.22977, −3.46562e+02, 2.87425e+03, −8.16148e+03, 6.93255e+03;...

  −7.05391, 2.81564e+02, −1.99672e+03, 5.25313e+03, −4.34765e+03];

if Fh<0.05

err = 1;

input(‘\n ERROR: \n \n Fh exceeds the lower value of 0.05 \n Press enter to continue: ‘);

elseif Fh> 0.54

input(‘\n ERROR: \n \n Fh exceeds the upper value of 0.54 \n Press enter to continue: ‘);

err = 1;

elseif Ft<0.59

err = 1;

input(‘\n ERROR: \n \n Ft exceeds the lower value of 0.59 \n Press enter to continue: ‘);

elseif Ft>0.98

err = 1;

input(‘\n ERROR: \n \n Ft exceeds the upper value of 0.98 \n Press enter to continue: ‘);

elseif load ~= [1,2]

err = 1;

input(‘\n ERROR: \n \n Insert a valid type of load, Press enter to continue: ‘);

else

err = 0;

end

end

if load == 1

  a = 9.2;

  b = 1.55;

  K = KP;

elseif load == 2

  a = 7.6;

  b = 0.952;

  K = KM;

end

Fta = [1; Ft^a; Ft^(2*a); Ft^(3*a); Ft^(4*a)];

Fhb = [1; Fh^(1/b); Fh^(2/b); Fh^(3/b); Fh^(4/b)];

kt = Fta’*K*Fhb

% ===================================================================

% ===================================================================
